# The Neural Basis of and a Common Neural Circuitry in Different Types of Pro-social Behavior

**DOI:** 10.3389/fpsyg.2018.00859

**Published:** 2018-06-05

**Authors:** Jun Luo

**Affiliations:** Neuro & Behavior EconLab, School of Economics, Center for Economic Behavior and Decision-Making, Zhejiang University of Finance & Economics, Hangzhou, China

**Keywords:** pro-social behaviors, neural basis, neural circuitry, functional magnetic resonance imaging, transcranial direct current stimulation

## Abstract

Pro-social behaviors are voluntary behaviors that benefit other people or society as a whole, such as charitable donations, cooperation, trust, altruistic punishment, and fairness. These behaviors have been widely described through non self-interest decision-making in behavioral experimental studies and are thought to be increased by social preference motives. Importantly, recent studies using a combination of neuroimaging and brain stimulation, designed to reveal the neural mechanisms of pro-social behaviors, have found that a wide range of brain areas, specifically the prefrontal cortex, anterior insula, anterior cingulate cortex, and amygdala, are correlated or causally related with pro-social behaviors. In this review, we summarize the research on the neural basis of various kinds of pro-social behaviors and describe a common shared neural circuitry of these pro-social behaviors. We introduce several general ways in which experimental economics and neuroscience can be combined to develop important contributions to understanding social decision-making and pro-social behaviors. Future research should attempt to explore the neural circuitry between the frontal lobes and deeper brain areas.

## Introduction

Humans are the most successful species at restraining their self-interest motives, even in interactions with unfamiliar strangers, through the development and enforcement of social norms (Fehr and Fischbacher, 2003; Boyd and Richerson, 2005). This human behavioral feature is thought to be a social adaptation that underlies our evolutionary success (Hrdy, 2009; de Waal, 2010). Pro-social behaviors, in particular, play a crucial role in social life across many cultures (Henrich et al., 2001). They represent a broad category of acts that are defined by significant regions of society as generally beneficial to other people or one’s group (Penner et al., 2005). Pro-social behavior involves trade-offs between our own well-being and the well-being of others, including a donation to charity, reciprocal exchange, interpersonal trust, mutual cooperation, costly punishment of norm violations. Pro-social behaviors that are exhibited in game tasks have been found and replicated under controlled environments in many behavioral experiments; players like to share wealth with strangers (Eckel and Grossman, 1996; Fehr and Fischbacher, 2003), punish defectors at a cost (Fehr and Gächter, 2002; Fehr and Fischbacher, 2003; Dawes et al., 2007), invest money in a stranger (Berg et al., 1995; Kosfeld et al., 2005) and reject unfair divisions of a sum of money (Güth et al., 1982; Camerer, 2003).

In this paper, we review studies on the neural activity of going against pure self-interest behaviors. This evidence is based on neuroimaging and brain stimulation approaches that provide a micro-foundation of pro-social behaviors with regard to the underlying neural networks. These studies that involve social preferences are based on neuroscientific methods that include the neural networks and motivational forces involved in charitable donations, rejections to unfair divisions, punishments for non-cooperation behavior at a cost, or decisions to trust in an investment game. The combination of economic game models with modern neuroscientific methods enables researchers to investigate the neural mechanisms of pro-social behaviors and to advance theoretical models of how we make decisions in a social context.

There has been a gradual appearance of studies that reveal the mechanisms of action of social preferences on the brain’s reward system, the role that affective factors play in economic decisions, and the neural model of the capacity to infer an actor’s mental state during a strategic game. According to these neuroscientific findings, we thus propose an integrated model for a common shared neural circuitry for various kinds of pro-social behaviors, involving the theory of mind network, the reward system, emotion-related brain regions and prefrontal cortical areas. Indeed, this review would be a fruitful starting point for future studies on a model of the neural circuitry involved in pro-social behaviors, by describing the relationship between the behavioral patterns of social preferences and the empirically verified parameters of the brain model. This will bring about an improved model of social decisions and a better understanding of the nature of pro-social behaviors.

## Empathy/Charitable Giving

We can empathize with others, that is, understand and share their emotions, feelings, motivations without any exogenous emotional stimulation. This crucial phenomenon of human social interactions occurs in various situations. Prior work from cognitive and behavioral psychology reveals the complex emotion process of empathy, including cognitive appraisal, cognitive perspective taking, and affect sharing (Decety and Jackson, 2004; Lamm et al., 2007; Olsson and Ochsner, 2008; Hein and Singer, 2008).

In accordance with these studies, advances in neuroscience enable us to gain new insights into the neural basis of empathy (Eisenberg, 2000; Hoffman, 2001; Preston and de Waal, 2002; de Vignemont and Singer, 2006; Batson, 2009). First, neuroscientific experiments about empathy indicate that the same neural circuits underlying both affective and cognitive processes are activated when we have a feeling and when others have this feeling. Preston and de Waal (2002) proposed a neuroscientific model of empathy, which specifically states that attended perception to another person in an emotional state automatically activates the participant’s representation of that state and that activation of these representations are associated with autonomic and somatic responses.

Moreover, imaging studies have also investigated the brain activity of empathic responses in the field of touch, smell, and pain. Wicker et al. (2003) have performed a functional magnetic resonance imaging (fMRI) study that reveal the same brain regions are activated when observing a facial expression of disgust and when inhaling disgusting odorants. Keysers et al. (2004) have found that there are similar neural mechanisms involved when participants are touched and when they observe someone else being touched by objects. Another study has assessed the brain activity associated with empathy for pain (Singer et al., 2004, 2006). They indicated that activity in the anterior cingulate cortex (ACC) and anterior insula (AI) was observed when participants either felt pain or observed pain in someone else. These brain areas compose the affective pain circuits that represent our responses to pain and our understanding of how others feel pain. Further studies have investigated the temporal dynamics of the neural mechanisms underlying empathy for pain using event-related brain potentials (ERPs). These results showed that the early and late responses to empathy are separately adjusted by the situational reality of the stimuli, and these results support the hypothesis that empathy for pain consists of early emotional sharing and later cognition evaluations (Fan and Han, 2008).

In addition, responses in DMPFC regions while mentalizing with others who have similar and dissimilar thoughts and beliefs have also been shown to predict empathy (Zaki et al., 2009; Majdandžić et al., 2016). Neuroimaging studies have also examined the relations between activation in specific brain areas related to social preferences and self-reported empathy and willingness to help (Tankersley et al., 2007; Mathur et al., 2010; Powers et al., 2015), and found the correlations between the reflexive engagement of neural mechanisms of mentalizing and altruistic behaviors for monetary allocation and time spent helping others (Waytz et al., 2012). In fact, additional research has also demonstrated that the brain activation of brain areas involved in empathy predicts pro-social behaviors toward social exclusion (Masten et al., 2011) and that such activation occurs when participants make decisions to donate money to their family members (Telzer et al., 2011); thus, the neural basis of empathy during in tasks involving charitable donations has received much attention.

A prior attempt on the neural basis of giving showed that the mesolimbic reward system, including ventral tegmental (VTA) and striatal areas were both engaged by receiving money and by anonymous donations to charitable organizations, suggesting that giving has its own reward (Moll et al., 2006). Further study has clarified that there are different neural mechanisms for purely altruistic and warm-glow motives for charitable giving (Harbaugh et al., 2007). To test these two motives, researchers have assessed fMRI while participants played a dictator game in which participants were required to make decisions about whether to give money to a charitable organization. All the participants were randomly assigned to mandatory and voluntary conditions. In the mandatory condition, participants observed money being transferred tax-like to a charitable organization. In the voluntary condition, subjects could make transfers voluntarily to the charity. Similar neural substrates linked to reward processing were elicited while participants received money themselves, when they performed free transfers, and when they observed the charity receiving money. However, this neural activation was higher when charitable giving was voluntary rather than mandatory.

In another study, the motivational mechanisms of charitable giving were identified by multivariate decoding techniques (Tusche et al., 2016). Neural responses in the AI predicted affective empathy for beneficiaries, while temporoparietal junction (TPJ) activity was associated with the degree of cognitive perspective taking, suggesting that these distinct paths of social cognition and psychological mechanisms differentially lead to intraindividual and interindividual heterogeneities in charitable giving. Indeed, there was specific neural evidence of a correlation between individual differences in helpful decisions and the neural activation of AI, ACC, and TPJ (Greening et al., 2014), and neural mechanisms of individual differences in empathy and pro-social behaviors were further revealed by reinforcement learning theory (Lockwood et al., 2016). However, how affective empathy is linked to pro-social behaviors in charitable giving and the neural circuitry underlying empathy in terms of multi-faceted cognitive and emotional process remain poorly understand. Thus, one possible direction is to integrate various constructs of the neural mechanisms of empathy and provide connections between the neural responses to empathy and charitable giving in future studies.

## Fairness/Inequity Aversion

People tend to helped those who helped them, and to hurt those who hurt them. Consequences that represent such preferences are called fairness equilibria (Rabin, 1993). This fairness effect has also been recognized in formal theory models of reciprocal fairness (Rabin, 1993) and inequity aversion (Fehr and Schmidt, 1999), both of which assume that there is a trade-off between fairness and individual benefits. To examine decisions about fairness, an ultimatum game (UG) has been proposed (Güth et al., 1982) involving strategic interaction behaviors. As the hypothesis of self-interest motivation, the responder in the UG should accept any non-zero offer from the other party. The proposer can expect this self-interest response, and then will give a smallest non-zero offer to responder.

However, a number of studies have found that offers are commonly around 50% of the sum amount no matter of the total monetary, and lower than 20% of the total offers have more than 50% probability of being rejected (Güth et al., 1982; Roth et al., 1991; Bolton and Zwick, 1995; Henrich et al., 2001). Strong evidence indicates that many subjects reject low offers from proposers in the UG (Henrich et al., 2001; Camerer, 2003). It is thus clear that the actual decisions in the game do not agree with the behaviors of the model predicted to be driven by self-interest motivation, and neuroscience research has begun to provide evidence for the mechanism underlying these decisions in an UG.

An fMRI study first investigated the neural basis of response decisions in an UG (Sanfey et al., 2003). They found that unfair proposals elicited neural activity in brain regions involved in both the processing of cognition (DLPFC) and emotion (bilateral AI); these areas showed greater activation with an unfair offer that was subsequently rejected, whereas a greater response was seen in the DLPFC when an unfair offer was accepted. Further, there was significantly stronger activity in the AI when a participant received an unfair offer from another human compared to the same offer from a computer partner. Finally, the unfair offer was also related to heightened activity in ACC, and may imply the conflict between cognitive and emotion process in the response decision-making for the unfair offer of UG. Thus, receiving unfair offers in an UG was weakly associated with increased activity in these brain areas (see Gabay et al., 2014; Feng et al., 2015 for meta-analyses). Indeed, activation of the AI region involved in emotional arousal and measured as an autonomic index of affective status, indicated that skin conductance responses were stronger for unfair offers and related to the rejection rate of unfair offers in an UG (van’t Wout et al., 2006).

Compared to unfair offers in an UG, fair offers led to greater activation in the VMPFC region. Importantly, the choice to reject unfair transfers is associated with improved activity in the AI region (Tabibnia et al., 2008). The key role of the ventromedial prefrontal cortex (VMPFC) in response decisions involving fairness preferences of the UG is also supported by neural evidence (Koenigs and Tranel, 2007) that patients with brain injuries in the VMPFC reject unfair offers in the UG more frequently than healthy participants, implying that the cost of declining non-zero offers is of less concern in the response decisions of the UG when the VMPFC is damaged. An ERP study (Boksem and De Cremer, 2010) showed that medial frontal negativity amplitude was greater for unfair offers than fair offers. Moreover, this effect was shown to be the greatest for responders with high fairness concerns.

To distinguish the functions of different brain areas in response decision-making in an UG, Knoch et al. (2006) used repetitive transcranial magnetic stimulation (rTMS) to inhibit the activation of the right DLPFC (rDLPFC) when responders in an UG faced unfair offers and observed a reduction in responders’ willingness to reject unfair offers from proposers, which suggests that participants are more unable to resist the temptation to accept unfair offers from partners. However, participants did not change their judgment for such offers to be unfair after receiving rTMS, which reveals that the rDLPFC is crucial in implementing fairness-related decisions. In terms of transfer decisions from a proposer, another rTMS study indicated that reducing the activity of the right lateral PFC (rLPFC) led to a significant decrease in transfers in the UG, but neither the expected rejection from responders nor the fairness judgments were changed by rTMS (Strang et al., 2014). To modulate the neural excitability (activate or reduce) of specific regions, Ruff et al. (2013) employed transcranial direct current stimulation (tDCS) to demonstrate whether fairness-related decisions in the UG rely causally on neural activation of the rLPFC region. This study revealed that anodal tDCS in rLPFC caused transfers improvement significantly while cathodal tDCS to the rLPFC decreased transfers in the UG compared to sham stimulation. Together, these results provide strong causal evidence for the rLPFC in the implementation of fairness preference.

A pervasive notion in social science is that people have a preference to reduce inequality gaps in wealth distribution (Fehr and Schmidt, 1999). Studies have thus used inequality aversion to represent a fairness motive. To explore the tendency for inequity aversion in distributive decisions, participants performed in a distribution task (similar to UG) while scanning fMRI (Hsu et al., 2008). The experimental results suggested that the putamen encodes efficiency, whereas the insula represents inequity, and the caudate/septal subgenual area responds to a trade-off in efficiency and inequity. Strikingly, the choice about inequitable allocation was related to greater insula activity.

Neural evidence for preference of inequality aversion in distributive decision was also revealed by Tricomi et al. (2010). They have employed fMRI to demonstrate the existence of inequality aversion preferences in the brain. Inequality was created in experiments by recruiting pairs of participants and giving one of them an endowment. The participant who received the endowment showed greater neural reward activation while providing transfers to “other” rather than “self,” whereas the participants who did not receive endowment showed a significantly greater activation in reward areas while providing transfers to “self” rather than “other.” These results suggest that people are rewarded for reductions in the wealth gap, and the neural mechanisms of reward are strongly related to both advantageous and disadvantageous inequality. Civai et al. (2012) were more concerned with the differential roles of the AI and MPFC in equality versus self-interest in distributive decisions, especially for disadvantageous unequal offers and consequent rejections. The researchers found that the AI region was active during unequal offers, whereas the activity of the MPFC was negatively associated with rejection decisions. When inequity and efficiency were in conflict, participants showed greater activity in a simplified prefrontal network, including the rDLPFC, VMPFC, and the connectivity between them, according to fMRI signals (Baumgartner et al., 2011). Individual differences in inequity aversion were predicted by the blood-oxygen-level dependent (BOLD) signals of the amygdala (AMYG) during a resource-sharing task involving inequitable distributions to one’s self and others (Haruno and Frith, 2010).

Taken together, social interactions with inequitable outcomes are linked to neural systems, including the AI, AMYG and prefrontal cortex, that are associated with affective and emotional signaling that alter distribution decisions by modulating fairness perceptions. In addition, inequity may induce a punishment action; thus, the neural networks implicated in inequity aversion could lead to the decision to punish at a cost to the punisher. On the other hand, the preference for inequity aversion may reflect how the neural processes that conform to inequity detection are influenced and further processed through emotional circuitry. Converging evidence indeed suggests that decision tasks related to inequity normally activate brain regions involved in affective processing. Further studies are needed to determine how these signals transform the decision to punish.

## Costly Punishment

Across cultures, human always engage in individual costs in readiness to punish violators (Henrich et al., 2001; Fehr and Fischbacher, 2004; Bernhard et al., 2006), who propose an unfair offer during monetary allocation or take a self-interest strategy during a social exchange (Fehr and Gächter, 2002; Egas and Riedl, 2008). Why would humans punish defectors of universally maintained rules while diminishing their personal benefits? The view from evolutionary economics (Boyd et al., 2003; Bowles, 2009) indicates that human behavior in costly punishment has profound evolutionary foundation, and promoting pro-social behaviors, such as reciprocity and cooperation (Nakamaru and Iwasa, 2006; Rand et al., 2010; Rand and Nowak, 2013; Peysakhovich et al., 2014). These suggest that sanction at the cost of personal gain evolved as a spontaneous mechanism rather than as an intended or deliberate pattern; people thus feel satisfaction when punishing norm defectors. It is obvious that costly punishment brings a huge array of discusses about its behavioral mechanism, and start to focus on neural basis of costly punishment in recent years to further explain why we have willingness to costly punish.

A Neuroimaging research (de Quervain et al., 2004) first provided essential insight into the neural networks that shape such costly punishment actions. They designed a context of economic exchange in which investors transferred endowments to agents, but agents did not send back money to investors. This action of non-reciprocity was observed by a third party. Subjects could choose to punish these violators, and symbolic and effective punishments were available. Symbolic punishments did not influence the material benefits of the violator, while effective punishments did decrease the violator’s payoff. They used positron emission tomography (PET) to scan the third party’s brains while they confronted with the defection and determined the sanction. The neuroimaging results suggested that punishing defections effectively instead of symbolically activated the dorsal striatum (DS) region, which plays an important role in the processing of reward. Furthermore, subjects with higher activity in the DS were ready to pay more costs to punish. These findings proved the hypothesis that humans may achieve satisfaction from the action of punishing violators, even when this punishment causes a monetary loss to themselves.

To further assess this satisfaction through punishing defectors, another neuroimaging study using fMRI scanned the brain reward regions of participants during two-person economic game involving costly punishments (Strobel et al., 2011). They found that, indeed, brain reward areas such as the nucleus accumbens (NAC) and DLPFC were activated by the action of punishment. In addition, this activation was similarly affected by genetic variation of dopamine turnover during both first player and third party punishments. Overall, these results suggest that the interactive network of cognition, affect and motivation form the driving force in costly punishments.

A recent study has also investigated the brain mediation mechanisms during costly punishments based on the BOLD responses in related brain areas (White et al., 2014). Subjects showed greater modulation of BOLD signals based on the level of costly punishment in regions of the reward neural network, for example, the AI cortex and caudate, whereas subjects showed negative modulation of BOLD signals as a level of costly punishment within posterior cingulate cortex (PCC) and VMPFC regions. Converging evidence seems to indicate a transform via the reward circuitry in mediating costly punishment. In addition, the neurobiological determinants have been found an influence in decisions of punishing costly (Crockett et al., 2013). Manipulating the serotonin system of participants during economic exchange game alters the possibility of punishment through modulating the activity of striatum, indicating that serotonin may create the sensitivity threshold for punishment processing.

Some brain stimulation studies provide a causal evidence of prefrontal cortex regions on decisions of costly punishment through changing the activity of prefrontal cortex (van’t Wout et al., 2005; Knoch et al., 2006, 2007). Subjects have a lower propensity to punish unfair behavior at a personal cost when rLPFC activity is restrained compared with the sham condition (Knoch et al., 2007). Based on this result, it can be expected that distinctions in the brain functions of the prefrontal cortex could illustrate individual variations in the willingness to punish, that is, the higher the individual baseline level of rLPFC activity, the greater the punishment behavior performed by the individual. To demonstrate whether individual differences in the activity levels of the rLPFC region predict participants’ willingness to provide costly punishments to other people, a neuroscience study measured participants’ resting-state electroencephalography (EEG) activity (Knoch et al., 2010) before they executed punishments for unfair proposals. A positive relationship was found between resting alpha activity in the rLPFC and the likelihood of a costly punishment. It is well known that the bilateral LPFC was associated with implementing of self-control and cognition processes (Miller and Cohen, 2001; Knoch et al., 2006; Cohen and Lieberman, 2010).

Another brain stimulation study on sanctions (Buckholtz et al., 2015) combined rTMS with fMRI to verify the explicit role of the DLPFC in pro-social behaviors induced by blame and punishment. The participants reduced punishments for violation activities when their brain activity in the DLPFC was inhibited by rTMS, but these participants’ blameworthiness ratings were not influenced. The researchers also used fMRI to observe punishment-selective DLPFC region recruitment. These results indicated that these two aspects of decisions are neurobiologically dissociable and confirm a selective causal effect of the DLPFC on punishment behavior. Thus, brain stimulation to related brain regions has a significant effect on norm compliance induced by social punishment threats, whereas stimulation left beliefs of what the norm regulated and subject expectations about social sanctions unaffected.

However, perhaps it is still unclear what the fundamental driving force for neural responses in decisions for costly punishments is. Du and Chang (2015) concluded that three main cognitive and affective functions occur in costly punishment contexts that might have a crucial effect on activating neural regions, such as cost-benefit calculations, inequity aversions and social reference frames. The previous studies show that these three cognitive and affective functions have different neural circuitries underlying the complicated decision process of costly punishments. Furthermore, these neural mechanisms, involving distinct cognitive and affective processes, are likely to interact with one another during the decision to punish at a cost, and such interactions may lead to individual deliberations on the execution of this decision. Therefore, how to differentiate the neural circuitries of these cognitive and affective functions during decision-making in costly punishment is a key issue that needs to be solved.

## Cooperation

Humans often cooperate with each other in society, even with irrelevant strangers and people they will never meet again. This behavioral feature in human is considered as a social adaptation, implying human success in evolutionary progress (Hrdy, 2009; de Waal, 2010). Some behavioral studies focused on what motivation promoted evolution of human cooperation, such as costly punishment, altruistic rewarding and strong reciprocity (Fehr and Gächter, 2002; Boyd et al., 2003; Bowles and Gintis, 2004). Cooperation behavior has also been illustrated extensively in the economic exchange game, for example the prisoner’s dilemma game (PDG) (Sally, 1995). In the standard PDG, two players’ payoffs depend on interaction of their decisions. The player can get the most payoff if she or he choose to defect and the partner choose to cooperate, while the least to the player happens if she or he choose to cooperate and the partner choose to defect. In addition, mutual cooperation takes a modest amount to each player, whereas mutual defection leads to a lesser payoff to the two players.

Neuroscience methods combined with the paradigms of game theory have examined the neural basis of cooperative behaviors. In two neuroimaging studies (Rilling et al., 2002, 2004), it was revealed that the ventral stratum was activated when playing in mutual cooperation with a partner in a game, as compared to playing with a computer partner. Rilling et al. (2002) first employed fMRI to scan subjects when they played with a paired partner in a repeated PDG to explore the neural substrates of cooperative behavior. They found that the activity of related brain regions, such as NAC, rostral ACC, orbitofrontal cortex (OFC) and the caudate nucleus, involved in reward processing were associated with cooperative behavior, and a crucial role of the striatum in mutual cooperative behavior was demonstrated. Participants’ mutual cooperative behavior leads to a higher BOLD signal in the related neural network during a PDG but results in a lower BOLD signal in the same regions if the partner defects. In subsequent research (Rilling et al., 2004), the reward neural network was also activated during cooperation in a sequential PDG, and subjects showed higher anterior paracingulate cortex and posterior STS activity when playing with person rather than with a computer. Cooperation following the defection of a partner would be characterized as an action against one’s anticipation of the reciprocity norm and, thus, increase activation of the left AMYG and the bilateral AI (Rilling et al., 2008).

In another experimental paradigm, pairs of subjects were required to perform the same estimation task and received a monetary reward for right answer (Fliessbach et al., 2007). A higher activation of the ventral striatum was linked with the amount of reward earned by the subject, while a lower activation of the same area was linked with the amount of reward paid to the partner. That is, when people are assessed and rewarded by an identical standard, the ventral striatum activity is more closely related to personal relative earnings than payments to the partner. This finding indicates the likelihood that the striatal involvement in rewarding processes seems to vary depend on whether a social exchange was considered to be competition or cooperation. Similarly, when participants were asked to play with a partner competitively or cooperatively during a board game, differential brain regions were activated in two distinct patterns of interaction. The results showed that cooperation caused higher activity in the medial orbitofrontal cortex (MOFC) and anterior frontal cortex (AFC) compared to competition (Decety et al., 2004; Babiloni et al., 2007). However, whether and how these cortical regions are linked to the striatal activity involved in cooperation are currently unknown.

To further elucidate the neural mechanisms of cooperation, King-Casas et al. (2008) recruited subjects suffering from borderline personality disorder (BPD) to play an iterative social interaction game with healthy subjects. Healthy subjects exhibited a linear correlation between AI activity and both the amount of monetary payoff received from the partner and the magnitude of money sent back to their partner. In contrast, subjects with BDP only showed a relationship between AI activity and the amount of money repaid to the partner, not the amount of money received from their partner. These results are evidence that individuals with BPD show impaired AI activity that leads to an inhibition of their ability to benefit from mutual cooperation. Thus, the insula and the ventral striatum track the social interaction decision of the partner of whether to reciprocate cooperation, representing an encoding of the reward processes for the satisfaction gained through mutual cooperation (Sanfey, 2007). In addition, a computational model of social value was provided to predict individual cooperative behavior, which indicated that people receive a signal of social value reward for mutual cooperation (Fareri et al., 2015). This signal of social value was strongly associated with greater activation of the ventral striatum and MPFC, which suggests that this signal predicts cooperative behavior in an iterative social exchange game.

In summary, the implications and motivations behind pro-social behaviors in economic games have been widely discussed, and scans of related brain areas when people play social interaction economic games with a partner could reveal individual differences in cooperative behavior. However, why are people willing to cooperate with the other people in a game? What are motives driving this behavior for all humans? Whereas some economic games have been used mainly to investigate cooperative behavioral consistency (Yamagishi et al., 2013; Peysakhovich et al., 2014), manipulating different economic games with the same subjects could also enable researchers to isolate within-subject motives in order to more accurately examine the nature of cooperative decisions (Brañas-Garza et al., 2014). Studies that have used this methodology have indicated that cooperative behavior is always multi-determined and can be assigned to completely different motives.

Prior work using imaging tools such as fMRI have allowed the identification of the neural networks involved in cooperative behavior. Nonetheless, these tools can provide only limited support to this ambitious purpose as they lack temporal resolution. In addition, they do not permit an on-line, real-life social exchange environment. However, social interaction is an essential part of cooperative behavior. Therefore, how our brains specifically exploit social cues and contexts when considering whether to cooperate remains unclear (Jahng et al., 2017). To account for the complexity of this event, the hyperscanning approach supports a high temporal resolution that allows the capture of simultaneous recordings of brain activity as a possible research direction.

## Trust/Trustworthiness

It is well known that trust penetrates into many aspects of our life, including working relations, friendships, and family relations. Interpersonal trust is also a core element for deeply understanding economic among people and the loss of trust between exchange partners seriously hinders market exchange. Thus, there are many reasons for researchers to concern the decision of trust. To investigate the decision of trust in economic interaction, Berg et al. (1995) firstly constructed a trust game, in which a player (the investor) has to decide how many amounts of endowment to invest with the other player (the trustee), and then the trustee can choose whether to give back and how much money return to the investor. As the model hypothesis of rational and self-interested people, the trustee will never return money to the investor. The investor can expect this rational decision from the trustee, and should never invest any amounts of money with the trustee.

Despite the predictions of game theory, in fact, most of the investors are still quite willing to transfer considerable amounts of money to a partner, and the trustees often repay some amount of money to the investor. Extensive studies have also discussed the potential factors that induce both trusting and trustworthiness behaviors among people that are not consistent with the hypothesis of the Homo economicus (Cook and Cooper, 2003; Bohnet and Zeckhauser, 2004; Cox, 2004; Ashraf et al., 2006; Schechter, 2007). In laboratory experiments, the subjects robustly showed behavior of trust although with completely strangers, or even when reputation is absent (McCabe and Smith, 2000; King-Casas et al., 2005).

Based on the results of behavioral studies, neuroscientists have attempted to provide the neural basis of trusting behavior. Krueger et al. (2007) employed hyper fMRI to scan pairs of subjects while they were playing against each other in a trust game. According to the within-brain and between brains analyses, several lines of evidence from functional brain activity indicated that the differential activation of related neural systems involves two trust strategies. First, the paracingulate cortex region is linked with the building of a relation of trust by inferring the partner’s intentions to predict the subsequent decision. Second, the more recently evolved brain areas could be distinctly involved in interactions with more primitive neural systems developing conditional and unconditional trust relations. Conditional trust decisions significantly activated the VTA region, associated with the estimation of expected rewards, while unconditional trust decisions activated the septal region, associated with social interaction behaviors.

Interestingly, the evidence from neuroendocrinology shows that humans can secrete two hormones in opposite ways that are linked to establish a subtle balance in adjustable trust behaviors. A hormone that promotes social trust is oxytocin (OT). There is evidence that the brain differentiates between interpersonal trust and risk-seeking, derived from a study where the synthetic neuropeptide OT was injected intranasally to subjects while playing a trust game (Kosfeld et al., 2005). The hypothesis was that the betrayal aversion reducing effect of OT might result in decreased activity in the AMYG, suggesting that OT reduces AMYG activity. AMYG function has been demonstrated to be involved in evaluating the trustworthiness of faces (Winston et al., 2002; Adolphs et al., 2005) and ambiguous incidents (Hsu et al., 2005), which both have relevance with decisions in a trust game. It should be noted that these effects of OT might not extend to all people, because a neuropathological study found that OT can inversely inhibit trust behavior in individuals with BPD (Bartz et al., 2010; Bos et al., 2010). These results demonstrate the necessity of taking into account personal heterogeneity while reporting the effects of hormones on individual behaviors (Bartz et al., 2011).

Similarly, in a testosterone administration and placebo-controlled experiment, Bos et al. (2012) used fMRI to provide insights into the neural mechanisms involved in the effect of testosterone on trusting behavior. They found that testosterone improved social vigilance to untrustworthy faces by affecting neuropeptide systems in the central AMYG region, enhancing the communication between the AMYG and brainstem areas. However, testosterone can also change the functional connectivity between the OFC and AMG while judging unfamiliar faces, which then induces an improvement in social vigilance by decreasing top–down control over the AMYG. Although speculative, a neurobiological interpretation based on these results is that testosterone leads to the continuous reduction, in an uncertain social interaction, of the connectivity between the OFC and AMG via a prefrontal-dopaminergic mechanism, which results in more vigilant AMYG responses to signals of untrustworthiness.

Other studies have specifically examined the neural basis of trustworthiness of trustees, and the mechanisms involved in decision factors such as risks, benefits, and reputation have been examined (Baumgartner et al., 2009; Knoch et al., 2009; van den Bos et al., 2009; Aimone et al., 2014). Specifically, researchers have paid attention to the correlation between altruism and trustworthiness in neuroscience studies. A clinical lesion example indicated that patients with injuries to the VMPFC region offer less in a dictator game and show less trustworthy behaviors in a trust game, suggesting that the VMPFC plays an indispensable role in both altruism and trustworthiness decisions (Krajbich et al., 2009; Moretto et al., 2013). There was evidence that the trustee playing the game showed activation in the VMPFC, the posterior cingulated cortex (PCC), the lateral OFC, and the right AMYG, and that the VMPFC response was linked with altruistic behavior (Li et al., 2009). Previous studies in neural cognition have also illustrated that the VMPFC is crucial for evaluating social information and that impairments in the VMPFC caused serious disruptions of emotion and resulted in impaired to decision making, behavior regulation and planning (Damasio, 1994; Anderson et al., 2006).

In addition to the VMPFC region, the roles that other brain areas play when realizing a partner’s trustworthiness have also been tested in other studies. In one study, the caudate nucleus activity predicted whether the trustee showed trustworthiness for a partner in a trust game (King-Casas et al., 2005). In a second study, researchers used the same paradigm of a trust game to investigate specializations of the cingulate cortex in encoding trustworthy decisions in a social domain (Tomlin et al., 2006). A further study arranged investors in a trust game to sequentially face three trustees and provided profiles that made them seem morally positive, neutral or negative in order to instill a prior belief about trustworthiness (Delgado et al., 2005). The researchers found that the caudate nucleus activity in the investors was involved in the decision of whether the trustees were weakened when the investors depended on the trustees’ information about moral character. Irrespective of the exact neural mechanisms, fMRI results indeed indicate that the AMYG is also associated with facial detections of trustworthiness (Winston et al., 2002; Engell et al., 2007; Todorov et al., 2008a,b; Said et al., 2009), and patients with damage to the bilateral AMYG show more facial evaluations of trustworthiness compared to healthy participants (Adolphs et al., 1998). The ACC and insula are also responsible to the processing of interpersonal trust and social threat (Rushworth et al., 2007).

Prior neuroimaging studies on trustworthiness have led to a well-founded discussion about the correlation between trustworthiness and empathy (Adolphs, 2002; Winston et al., 2002; Engell et al., 2007; Said et al., 2009). In particular, two meta-analyses studies have summarized the differential neural circuits linked to the process of identifying trustworthy and untrustworthy faces (Bzdok et al., 2011; Mende-Siedlecki et al., 2013). Notably, faces considered to be trustworthy primarily involve activity in reward-related brain areas, while faces considered to be untrustworthy primarily engage activation of the ventral AMYG, which quickly responds to a potential threat. A recent ERP study showed that faces perceived as trustworthy are implicitly evaluated even during an unrelated task, as when subjects must memorize characteristics, as seen by the modulation of neural activity linked with visual working memory that processes faces (Meconi et al., 2014).

However, these neuroimaging studies have failed to provide a direct causal effect between the activity in related brain areas and behavioral decisions. In contrast, recent tDCS studies, by affecting brain activity non-invasively, have established causal links between brain activity and trust or trustworthiness decisions. Colzato et al. (2015) used tDCS over the VMPFC region while participants played a trust game and did not found a correlation between VMPFC activity and trust behavior. Other tDCS studies showed that the modulation of activity in several brain areas, such as the OFC, DLPFC and VMPFC may change the subjects’ trustworthy behavior (Nihonsugi et al., 2015; Wang et al., 2016; Zheng et al., 2016).

On the whole, these results provide insight into understanding how related brain areas work together when subjects exhibit reciprocal trusting by showing how these neural substrates are distinctly derived from reciprocated trust, betrayal aversion, risk preferences and perspective-taking motives. However, it is still not clear which mechanisms connect these neural substrates that underlie the different motivations in trust behavior, or which neural structures factors determine individual levels of trust behavior. Additionally, identifying the effect of social factors, such as social status, social information and peer influence, on trust behavior based on neural results is necessary in further studies.

In summary, we describe previous experimental studies that used neuroscience methods to explore the role of related brain areas in various pro-social behaviors (see **Table 1**).

**Table 1:** Summary of the study for the neural basis of pro-social behaviors.

Study	Technology	Experimental Task	Pro-social behaviors	Brain areas	Experimental design	Sample size
Rilling et al., 2002	fMRI	Prisoner’s Dilemma Game	Cooperation	Ventral striatum	Between (human versus computer) and within (iterated game) subjects.	36
Sanfey et al., 2003	fMRI	Ultimatum game	Fairness	AI and DLPFC	30 rounds in all, 10 playing the game with a human, 10 with a computer, and a further 10 control rounds.	19
de Quervain et al., 2004	PET	Third-party punishment game	Costly punishment	Dorsal striatum	Participants experienced four different conditions.	14
Rilling et al., 2004	fMRI	UG and PDG	Fairness	aPCC and posterior STS.	Between (human versus computer) and within (iterated game) subjects.	19
Decety et al., 2004	fMRI	Computer game	Cooperation	OFC and MPFC	Between (alone, cooperation, or against) subjects.	12
Moll et al., 2006	fMRI	Charitable donation	Altruistic behavior	VTA and STR	Different payoff types were designed: (*i*) pure monetary reward, (*ii*) non-costly donation, and (*iii*) costly donation.	19
Knoch et al., 2006	rTMS	Ultimatum game	Fairness	DLPFC	Applied rTMS to the right or to the left DLPFC and a control group.	52
Knoch et al., 2010	EEG	Ultimatum game	Costly punishment	rPFC	Responder played with 12 different proposers.	20
Spitzer et al., 2007	fMRI	Dictator game with the sanction threat	Costly punishment	OFC and DLPFC	Control and punishment conditions	45
Krueger et al., 2007	hyperfMRI	Trust game	Trust	pACC and VTA	Sequential decisions for monetary payoffs (low, medium, or high).	44
Emonds et al., 2011	fMRI	PDG and coordination game	Reciprocity	DLPFC and STS	Between (proself or prosocial) and within (two games) subjects.	28
Bos et al., 2012	fMRI	Rate facial pictures	Trustworthiness	OFC and AMYG	A randomized, counterbalanced, placebo-controlled, testosterone administration paradigm.	16
Ruff et al., 2013	tDCS	Ultimatum game	Fairness	rLPFC	Randomly assigned to one of three groups: anodal, sham, or cathodal.	64
Aimone et al., 2014	fMRI	Trust game	Betrayal aversion	AI	Both within- and between-subject	30
Strang et al., 2014	TMS	Dictator game	Strategic fairness	DLPFC	Randomly assigned to one of three groups: anodal, sham, or cathodal.	17
Zheng et al., 2016	tDCS	Trust game	Trustworthiness	VMPFC	Randomly assigned to one of three groups: anodal, sham, or cathodal.	60


## Neural Circuitries of Pro-Social Behaviors

**FIGURE 1:**
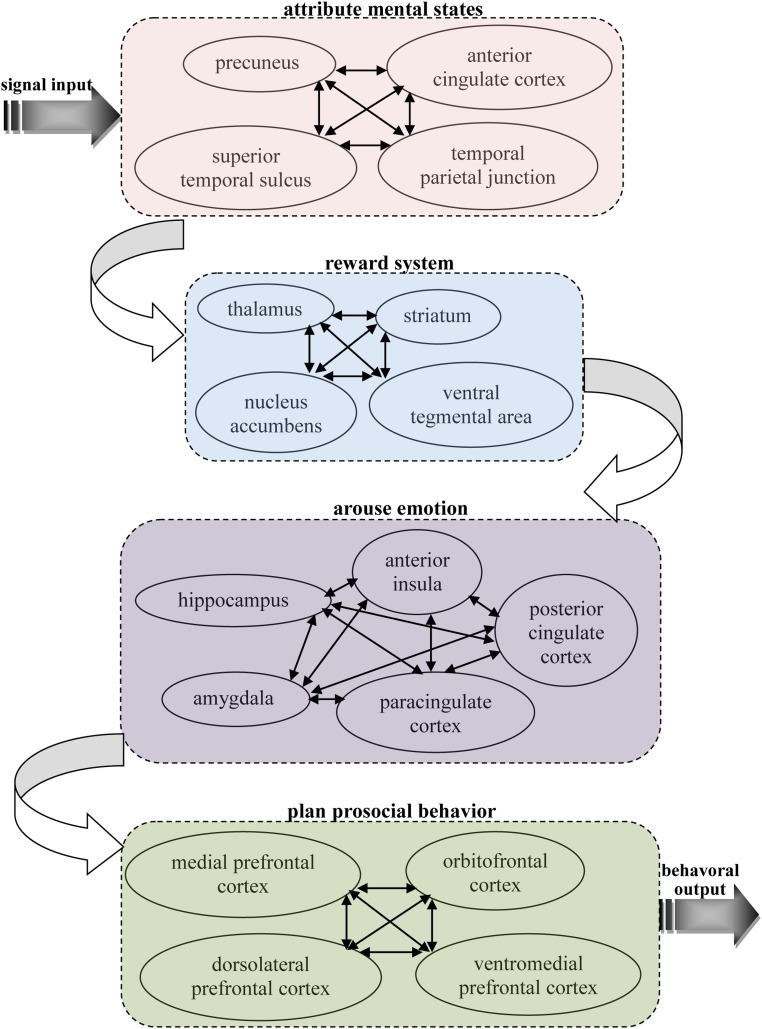
A possible common neural circuitry associated with different types of pro-social behavior in the human brain.

Based on previous neuroscience studies of different types of pro-social behaviors, it can be seen that there are some similar properties in the neural basis of these behaviors, which all activate related brain areas, including the theory of mind network, reward system, and prefrontal cortex. This implies there may be specific connections between the functions of these areas that lead to people often making decisions according to their other-regarding preferences. Our knowledge of this connection can help us better understand the neural mechanisms of pro-social behaviors. Thus, it is important to find the shared, common neural substrates that link these different types of pro-social behaviors. Here, an integrated model is proposed that depicts the neural circuits by which decision making is firstly primed in the theory of mind network while receiving input from other’s information, then the social cognition signal activates reward system, and this action then activates the brain areas associated with emotion to reinforce the reward experience, and, finally, the decision is reflected upon in the prefrontal cortex to execute a pro-social behavior (see **Figure 1**).

Pro-social behaviors surely requires theory of mind during cognition signal input, as it is the neural network underlying our ability to attribute other’s mental states, providing us with a prediction of their intentions and actions. Theory of mind is likely to provide our other-regarding, which allows the ability to share others’ thoughts and feelings and, therefore, motivates pro-social behaviors (de Waal, 2008). A number of studies in cognitive neuroscience have discovered the neural network for considering another person’s thoughts, comprising the precuneus, bilateral TPJ and right superior temporal sulcus (RSTS) (Rilling et al., 2004; Saxe, 2006; Young et al., 2007; van Overwalle and Baetens, 2009; Ye et al., 2015). In particular, the TPJ shows increased activity when participants read about a person’s beliefs in non-moral (Saxe and Powell, 2006) and moral (Young et al., 2007) contexts. Accordingly, it has also been suggested that the ACC might play an important role in representing the mental states of others (Gallagher et al., 2002; Gallagher and Frith, 2003; Rilling et al., 2004). This brain area is involved not only when mentalizing about the thoughts, intentions or beliefs of others but also when people are attending to their own states. Frith and Frith (2003) suggest that this area subserves the formation of decoupled representations of beliefs about the world. In addition, it has been shown that activity in this area is strongly associated with the level of an individual’s pro-social behavior (Tankersley et al., 2007). These results address long-standing discussions about the sources of social decision making by indicating that pro-social behaviors might first derive from the theory of mind network, with its the proclivity toward social-cognitive thoughts of other’s mental states.

Pro-social behaviors are always accompanied by the activation of the reward system, and these behaviors were marked and intensified after other-regarding thoughts of mental states. The reward system is a neural network responsible for incentive salience (i.e., craving, motivation, or desiring for a reward), related learning (mainly positive reinforcement for actions), and the activation of emotions, especially ones that involve pleasure as a central constituent (e.g., happiness, joy, and euphoria). Importantly, correlations between different kinds of pro-social behaviors and the reward system have been shown in many previous neuroscience studies. Moll et al. (2006) found that the reward system, such as VTA and striatum areas were both activated when participants give donations to the charity. A significant activation in the reward system in response to inequity aversion has also been found (Tricomi et al., 2010). Neuroimaging results suggest that punishment defection in an economic game activates the participants’ brain reward regions, such as the NACs and thalamus (de Quervain et al., 2004; Strobel et al., 2011). It was also demonstrated that the activities of related brain regions, such as NACs and the striatum area involved in reward processing were strongly correlated with cooperation behavior (Rilling et al., 2002, 2004; Sanfey, 2007). In addition, trust behaviors are primarily linked to reward-related brain areas when participants identify trustworthy faces (Meconi et al., 2014). These findings provide strong evidence of the rewarding process of different types of pro-social behaviors, which in return demonstrates the crucial role the reward system plays in the shared neural substrates of pro-social behaviors.

Pro-social behaviors inevitably activate emotion-related regions in the brain when these decisions are rewarded, and these reward experiences must be stored in memory. The emotion-related system is a group of neural structures that are primarily involved in many of our feelings and motivations, including fear and disgust. The AI is thought to process convergent information to create a relevant context for the emotions involved in a sensory experience. Certain structures in this system are involved in memory processing as well. The AMYG is responsible for determining where memories are stored in the brain and which the memories are stored. It is believed that this determination is based on how great an emotional response the action invokes. The hippocampus sends out memories to related brain regions for long-term storage. In sum, this system supports various functions, such as interpreting emotional signals, regulating hormones, storing memories and processing motivation. Therefore, it is easy to infer that these neural structures are related with pro-social behaviors. A recent study has also found that the BOLD signals from the AI predicted affective empathy and helpful decisions (Greening et al., 2014; Tusche et al., 2016), and the BOLD signals from the AMYG region can be used to assess individual differences in fairness (Haruno and Frith, 2010). In fact, neural responses in the AI region associated with emotional arousal through the measurement of an autonomic index in affective status indicated that there was a strong correlation between AI activity and inequity aversion (van’t Wout et al., 2006; Civai et al., 2012), and healthy participants exhibited a strong relationship between trusting behaviors and activity in this neural system including AI, AMYG, and PCC regions (Adolphs et al., 2005; Krueger et al., 2007; King-Casas et al., 2008; Watanabe et al., 2014). Bos et al. (2012) explored the neural mechanisms regarding the cause effect of testosterone on trusting behaviors via neuropeptide systems in the central AMYG region. In addition, it was shown that participants showed a significant modulation of neural responses within the PCC as a function of costly punishments and trusting behaviors (Li et al., 2009; White et al., 2014). Rilling et al. (2008) has also found that cooperative behaviors increase AMYG and AI activity, and subjects showed a higher anterior paracingulate cortex activity when playing cooperation strategies (Rilling et al., 2004). In conclusion, there is enough evidence to indicate that these neural structures, triggered by the need for arousing emotions and long-term memory play an important role in the production and reinforcement of pro-social behaviors.

Pro-social behavior truly needs to be controlled and planned as a whole while balancing various motivations, and the prefrontal cortex region is considered to be the control center of pro-social behavior and is the key part of the common shared neural substrates of different types of pro-social behaviors. The prefrontal cortex region is known to be associated with planning and modulating pro-social behaviors (Yang and Raine, 2009). In terms of psychology, the most typical functions carried out through the prefrontal cortex are executive functions (Shimamura, 2000). Executive functions involve the abilities to make decisions among different conflicting considerations (Goldberg, 2002); determine good or bad, self-interest or other-regarding, and same or different; create expectations according to events; create predictions of consequences and future outcomes of current actions; and enable social “control” (the capability to inhibit desires that, if not inhibited, could cause socially unacceptable consequences). The effect of this neural network on carrying out pro-social behaviors has been demonstrated in many neuroscience studies. For example, several studies have shown that individual differences in signals from the MPFC are correlated with empathy and altruistic behaviors (Zaki et al., 2009; Wagner et al., 2011; Powers et al., 2015). The indispensable roles of the VMPFC in the decision making involved in fairness, altruism and trustworthiness are also supported by previous neural evidence (Koenigs and Tranel, 2007; Krajbich et al., 2009; Moretto et al., 2013). A fMRI study (Spitzer et al., 2007) investigated the relationship between costly punishments and activity in the lateral OFC and rDLPFC and the causal effect of activity in the LPFC region on decisions of costly punishment by altering the activation of this region (van’t Wout et al., 2005; Knoch et al., 2006, 2007). Interestingly, other brain stimulation studies have indicated that reducing the activation of the rLPFC leads to a significant change in fairness-related behaviors, but neither expected punishment from others nor fairness norms were altered (Ruff et al., 2013; Strang et al., 2014). These findings reveal that activity in the prefrontal cortex is a crucial biological prerequisite for an important aspect of evolutionary and social human behavior. These findings suggest that the prefrontal cortex is responsible for behavioral control although it is dissociated from the neural structures that enable people to anticipate social norms and attribute other’s mental states. The structural connectivity and situation-dependent functions of prefrontal cortex (Duncan, 2010) make it possible to integrate and coordinate activation of the neural networks related to pro-social behavior during action control.

## Discussion

This review introduces several widely used methods that combined the game theory of economics with neuroscience technologies to develop new insights into understanding pro-social behaviors. These findings contribute important progress for measuring the neural mechanisms involved in pro-social behaviors and yield the guarantee of identifying and accurately characterizing both the mechanisms and the factors that influence interactions and engagements in pro-social behavior.

The economic game approach has several some advantages over typical paradigms of decision-making, not only for use within real, sequential, social interactions that make it possible to study complex exchange contexts such as fairness, trust, cooperation, and norm compliance. On the other hand, neuroscience methods of assessing pro-social behavior have obtained several prominent achievements in examining how the utility of parameters of behavior are represented in neural systems (Knutson et al., 2005; Sugrue et al., 2005; Padoa-Schioppa and Assad, 2006). Thus, neuroscience studies of pro-social behaviors could explore the neural circuitries of parameters that game models both expect (such as strategic payoffs) and do not expect (such as social preference). In addition, behavioral and neural data created through this method can confirm significance in offer special restraints, based on neural networks, for any theory that attempts to build precise models of pro-social behavior.

However, there are important challenges to address for any novel approach. These challenges are involved in disciplines that manipulate diverse analysis perspectives and have distinct theoretical assumptions. In particular, there are crucial differences in study methodologies, such as the use of deception, which is strictly prohibited in economics but widely used in neuroscience and psychology. Furthermore, it is important to be prudent when understanding brain activity measured by neuroimaging methods. For instance, the correlation of a brain area with either reward encoding or emotion processing in prior studies does not necessarily mean that this brain region’s activity during at social interaction game can automatically be considered, respectively, as involved in reward or punishments (Sanfey, 2007). Therefore, one should be cautious in this field and support these conclusions by collecting evidence from other methodologies or, at a minimum, illustrating that behavioral results are consistent with the identified neural activities, for example, high levels of activation of the rewarding neural network being associated with an individual’s preference for social decisions (de Quervain et al., 2004).

Although neuroimaging data cannot provide causal inferences, it is likely close to causality by predicting decision making during a treatment based on brain region activity during another treatment. For example, individual heterogeneity in the activity of the caudate nucleus when punishment is free predicts the level of willingness to punish when the punishing is costly (de Quervain et al., 2004). Similarly, individual differences in the activation of striatal regions when donations are mandatory are associated with participants’ willingness to give money when this is a voluntary behavior (Harbaugh et al., 2007). These results further provide evidence of the rewarding process of pro-social behavior, which in return supports the hypothesis of shared neural circuitries of social reward and other primary and secondary reward (Montague and Berns, 2002).

Future studies in neuroscience should exploit the wide range of available tools, by using multiple measurement methods simultaneously (such as fMRI and rTMS, tDCS, or hormone measurement) along with the valuable behavioral parameters and theoretical predictions from complex game models. It is well known that non-invasive brain stimulation can establish causal correlations between related brain activities and individual behaviors by altering these neural processes and subsequent individual behaviorally expressed preferences. These methods have been used to not only support a biological basis for a mathematical characterizations in pro-social behaviors that are based on neural systems but also provide predictions of brain activity in social interactions and economic exchanges and how these behaviors can be transformed when manipulated by rTMS, tDCS, and other tools.

Nevertheless, brain stimulation technology is not the only approach to establish a causal inference between identified neural circuits and individuals’ pro-social behaviors. Several pharmacological studies show great potential in this domain. For example, testosterone can increase the fairness transfers of a proposer (Eisenegger et al., 2010) and the probability that a responder rejects unfair offers (Burnham, 2007) in an UG; the neurohormone oxytocin improves behaviors of trust but not trustworthiness (Kosfeld et al., 2005); the depletion of the neurotransmitter serotonin increases the number of rejection decisions for unfair offers in an UG (Crockett et al., 2008; Crockett, 2009); and benzodiazepine can decrease the number of decisions to reject (Gospic et al., 2011). These studies of pharmacological interventions combined with fMRI allow observations of how neural systems causally influence behavioral changes (Baumgartner et al., 2008; Gospic et al., 2011).

In addition, several neuroscience studies have began to consider how the neural systems involved in pro-social behaviors are influenced by various factors. One factor is “social image,” that is, how does knowing that other people are watching you influence your decisions and brain activity? This topic has been a concern of economists (Andreoni and Bernheim, 2009) and is important because social image could be changed by different organizational norms in information and institutions. An fMRI study indicated that the bilateral striatum was more highly activated when participants’ charitable donations were observed than in the control treatment (Izuma et al., 2008), which supports the hypothesis that a social image rooted in charitable giving is rewarding. Consistent with a wide range of inequity aversion, one study was concerned with whether an awareness of high-status people suffering a failure would create a positive reward. Activity in the ventral striatum was found in response to these hypothetical contexts, and the BOLD signal predicted self-rated decisions (Takahashi et al., 2009). Emotions can also have an impact on pro-social behaviors. A neuroimaging study exploring this topic was based on real crime cases with “mitigating circumstances” (Yamada et al., 2012) and found that the activity in the insula, an identified neural correlate of empathy, was related to the level of sentence reduction.

However, our current understanding of neural mechanisms of pro-social behavior is still limited. This understanding will be improved if we obtain additional interpretations of the genetic and neurophysiological mechanisms of information processing in neural reward systems. Previous studies have revealed that social reward generally activate the ventral or DS, and there is a substantial overlap between the activity in these regions and the activity observed in studies about anticipated monetary reward or reinforcement learning (Fehr and Camerer, 2007; Fehr, 2009). This overlap is in accordance with the hypothesis that social preferences are similar to preferences for physical reward in terms of brain activity, which supports the theory about which decisions reflect a tradeoff between one’s own benefits and the benefits of others.

More importantly, our brain has to compare social welfare and individual benefits and solve a conflict between these aspects when we exhibit pro-social behaviors. Previous studies have demonstrated that the prefrontal cortical regions that evolved lately (in evolutionary perspective) play crucial roles in this process of conflict resolution. For example, the VMPFC region is more activated when subjects can punish defectors at a personal cost than when punishment is free (de Quervain et al., 2004). Both the VMPFC and dorsal ACC regions show high activation levels when participants give charitable donations involving a cost (Moll et al., 2006). The ACC is known that have an important effect on conflict monitoring (Botvinick et al., 2001), so the activation of this area aligns with the presence of a conflict between pro-social motivations and self-interest incentives. In addition, the value of the response of the VMPFC is influenced by other responses of the posterior superior temporal cortex (PSTC) that have been shown to be crucial in overwhelming egocentricity prejudice, suggesting that the activity in both the VMPFC and the PSTC are important constituents of the neural network of pro-social behaviors.

In addition, the crucial role of DLFPC region in the processing of pro-social behavior has also been demonstrated (Sanfey et al., 2003). This study investigated the neural networks involved in the response decisions of an UG in which a rejection of unfair transfers indicates a balance between a self-interest motive and a fairness motive. Indeed, a function of the DLPFC may be enabling an individual to make choices for their long-term benefit for a good reputation in social interactions rather than the short-term benefit of the individual (van den Bos et al., 2009). The effect of the DLPFC on overwhelming short-term self-interest has also been investigated, and results show that the behavior of compliance with norms under the threat of punishment is positively related with the level of activity in the DLPFC (Spitzer et al., 2007).

More importantly, neuroscientists have indicated that we cannot consider brain regions as separate mini-brains but rather as widely interconnected regions. Notably, the frontal lobes are more linked to other brain regions than any other parts of the brain (Goldberg, 2002). In addition, the frontal lobes perform the most complex and developed functions of all parts of the brain, namely, the executive functions. This region is involved in complex, purposeful, and intentional decision making. However, previous studies also show that other brain areas, especially activation of brain–stem structures and the prefrontal cortex, are commonly associated with pro-social behaviors, such as the AI and the rostral ACC, which are activated when subjects empathize with other people experiencing pain (Singer et al., 2004); the ventral striatum, whose activity is strongly correlated with the amount of money given to charity (Harbaugh et al., 2007); the amygdale, whose BOLD signals can predict individual differences in aversion to inequity (Haruno and Frith, 2010); and both the DLPFC and caudate nucleus, whose activities are high when individuals receive unfair transfers from partners (Harbaugh et al., 2007). We also propose a common shared neural network of pro-social behaviors involving the theory of mind network, emotion-related regions, the reward system and the prefrontal cortex. In this neural circuitry, the control function of the prefrontal cortex plays a key role in coordinating human rationales, emotion, perception, cognition, motivation and reinforcement learning.

Among all species, humans are unique in terms of the ways in which they govern social life by executing pro-social behaviors. In addition to having the longer period during which brain development has been shaped by living environment, human beings change their environment, which shapes brains to an unprecedented extent among other species (Wexler, 2006). We thus suggest that the evolutionary consequence of promoting pro-social behaviors is the development of the prefrontal cortex, which plays a crucial role in the neural circuitry of social preferences. The prefrontal cortex connected with other brain regions when executing social cognition functions, but the region-to-region interaction mechanisms between the prefrontal cortex and deeper brain areas that represent pro-social behaviors are still not clear.

Finally, the overall target of this effort is the identification of a complete and general model of the neural network of decisions involved in pro-social preferences. There have been previous attempts from both “cognitive neuroscience in social decisions” and “neuroeconomics” to interpret the social brain and the related moral emotions (Adolphs, 2001, 2003; Greene et al., 2001; McCabe et al., 2001; Moll et al., 2002; Rilling et al., 2002; Sanfey et al., 2003; Singer et al., 2004). In addition, a paradigm with high sensitivity may indicate how affective and cognitive responses in the brain diverge or converge throughout decision making. Future studies combining fMRI with another method with a higher temporal resolution, such as EEG or functional near-infrared spectroscopy (fNIRS), may describe novel data on the region-to-region interactions between neural activities associated with cost-benefit calculations, social preferences, and the processing of information across self and others. Interestingly, finding a non-human primate model for pro-social behaviors can complement studies in humans by demonstrating specific neuronal circuitries in the core neural processes using single-unit recordings and pharmacological interventions in particular populations of neurons. Another important aspect of concern in the neuroscientific study of pro-social behavior is the social context in which pro-social behavior occurs. Understanding how the social context transforms the neural processes involved in cost-benefit calculations, social preferences, and the process of information between self and others will lead to better interpretations of the complex events behind human pro-social behaviors.

## Author Contributions

JL drew the table, wrote and revised the manuscript, and finally approved the version to be published.

## Conflict of Interest Statement

The author declares that the research was conducted in the absence of any commercial or financial relationships that could be construed as a potential conflict of interest. The author alone is responsible for the content and writing of this paper.
